# Be Sanitary and Be Sane

**Published:** 1899-10

**Authors:** 


"Be Sanitary and Be Sane."—This is the motto adopted by the Sanitary Association of Citizens at San Antonio. It is to no purpose to suggest that they have the cart before the horse, and should say "Be sane and you will be sanitary"; that is, all "sane" persons (persons of sound mind) are expected to be "clean," cleanly; take measures to insure health, etc. There is a shaking up of dry bones in the Alamo City, and we expect to be able shortly to show a big decline in the death rate.

Attention is called to Mr. Braden's letter in this issue. The Plumbers' League are working energetically with the masses, and are having the support of the secular press, and it is hoped that much good will result from enlightening the people on the dangers that they are subject to by reason of defective or unscientific plumbing. Mr. Braden says, in support of the law regulating plumbing, that it is as rational as the law regulating the practice of medicine. Well, now, that is an unintentional sarcasm, seeing that the great State of Texas has never seen fit to put any restrictions whatever upon this, the most dangerous privilege that can be entrusted to a person. It looks like a paradox that our wise men should have been made to see the dangers to public health resulting from an indiscriminate and unenlightened practice of plumbing, and yet deliberately shut their eyes to those still greater dangers resulting from allowing any ignoramus to "practice medicine" (as alleged); to entrust human lives and health and happiness to any fellow who feels that he is "called to practice," and can show a diploma from anywhere, signed by anybody or nobody. A diploma is "evidence of a degree having been conferred" (Webster), and "conveys no rights or privileges whatever" (Webster and numerous court decisions). Nevertheless, Texas will and does accept, as LICENSE to practice, a "diploma" from anywhere. Under some State laws, Texas included, any five men may associate themselves, call themselves the "faculty," and for $10 procure a charter to run a MEDICAL COLLEGE, and its diploma is accepted in Texas as a license to deal with life and death. What a burlesque on civilized government! Meantime, for want of intelligent action, such sanitary outrages may be perpetrated on the people as the emptying of sewers into the streams; at Austin, notably. Here we have a city of 28,000 people whose sewers are owned by *private individuals,* and are allowed to discharge their disease-producing human excreta and all other filth into the beautiful Colorado, whose banks are lined with villages of happy and unsuspicious people who drink its water. But the crowning piece of jackassism in this line was permitting five thousand troops and one thousand horses to camp, two months, during the rainy season, on the hills overlooking the great lake, the dam of which had cost Austin nearly $2,000,000, and built to store drinking water for the people, and where all the scourings of the camp, and the flushings (by the rains) of the privies necessarily drained into our drinking water. Such ignorance and stupidity would have shamed the dark ages.

THE LAW REGULATING PLUMBING, to which reference has been made, is primarily in the interest of the Plumbers' League, to protect them from cheap and worthless competition; secondarily in the interest of public health. It is, nevertheless, a good law, and should be enforced. But one fatal defect in it renders it worthless. It makes it the duty of all towns of ten thousand and over to appoint boards of examiners to examine and license plumbers, and only those who can pass examination are allowed, under penalty, to follow the calling. If a city shall neglect to do this thing, there is no way to compel it, and like Austin, they just will not do it, because certain rich men have boys doing plumbing work, and who will not guarantee even the stopping of a leaky faucet, as one of said boss plumbers (alleged) told yours truly yesterday when he paid him for a boy's time who went twice to stop a leaky faucet and didn't do it at last. The wise men of the Legislature always claimed that a law to regulate the practice of medicine would be "class legislation." What would a man-up-a-tree call the plumbers' law?

